# A Socio-Ecological Framework for Cancer Control in the Pacific: A Community Case Study of the US Affiliated Pacific Island Jurisdictions 1997–2017

**DOI:** 10.3389/fpubh.2018.00313

**Published:** 2018-11-13

**Authors:** Neal A. Palafox, Martina Reichhardt, John Ray Taitano, Mavis Nitta, Helentina Garstang, Sheldon Riklon, Livinson Taulung, Lee E. Buenconsejo-Lum

**Affiliations:** ^1^Department of Family Medicine and Community Health, John A. Burns School of Medicine, University of Hawaii, Honolulu, HI, United States; ^2^Yap State Department of Health Services, Yap, Micronesia; ^3^Cancer Council of the Pacific Islands, Tamuning, GU, United States; ^4^Republic of the Marshall Islands Ministry of Health, Majuro, Marshall Islands; ^5^Department of Family and Preventive Medicine, University of Arkansas for Medical Sciences Northwest, Fayetteville, AR, United States; ^6^Kosrae State Department of Health Services, Kosrae, Micronesia

**Keywords:** pacific, cancer, disparities, coalition, social ecology, management, Guam, Micronesia

## Abstract

The United States Affiliated Pacific Island Jurisdictions (USAPIJ) are politically associated to the United States (US) as US Territories (Guam, American Samoa), a US Commonwealth (Commonwealth of the Northern Mariana Islands), and as sovereign nations linked to the US through Compacts of Free Association [Federated States of Micronesia (FSM), Republic of the Marshall Islands (RMI), Republic of Palau (ROP)]. Cervical cancer incidence in the RMI is the highest in the world, mammography services are not available in the FSM and only Guam has on-island oncology services. Cancer risk factors such as obesity, tobacco, and Hepatitis B are prevalent. Twelve years of nuclear testing in the RMI adds to the cancer burden. A community-based, multi-national coalition with multi-system external partners the Pacific Regional Cancer Control Partnership (PRCP) was developed to address cancer prevention and control in the USAPIJ. Through the PRCP, local cancer coalitions, a regional cancer registry, 12 years of regional cancer control plans, and cancer prevention programs and research has been implemented.

**Methods:** The PRCP is the subject of this community case study. The PRCP is analyzed through a socio-ecological theoretical framework to contextualize its typology, building blocks, and management. The respective roles and work of each partner and organization will be described and aligned with the levels of the socio-ecological framework.

**Results:** The USAPIJs evolved a community-focused internal and external regional cancer prevention and control network over 20 years. The function and structure of the PRCP fits within a socio-ecological framework for cancer control. An adaptive management strategy has been used within the PRCP to manage its multi-national, multi-level, and multi-system partners.

**Conclusion:** The PRCP has been able to advance cancer prevention and control programs with a community-centric model that functions in a multi-national, multi-cultural, low-resource, geographically dispersed environment over the last 20 years. The PRCP operates with a structure and management style that is consistent with a socio-ecological framework for cancer control. This case study provides a blueprint for the PRCP organizational structure and a mechanism for its function. The PRCP concept, a community-centric model for cancer control in multi-national resource-limited environments, may be scaled to other global environments.

## Introduction

**Figure 1:**
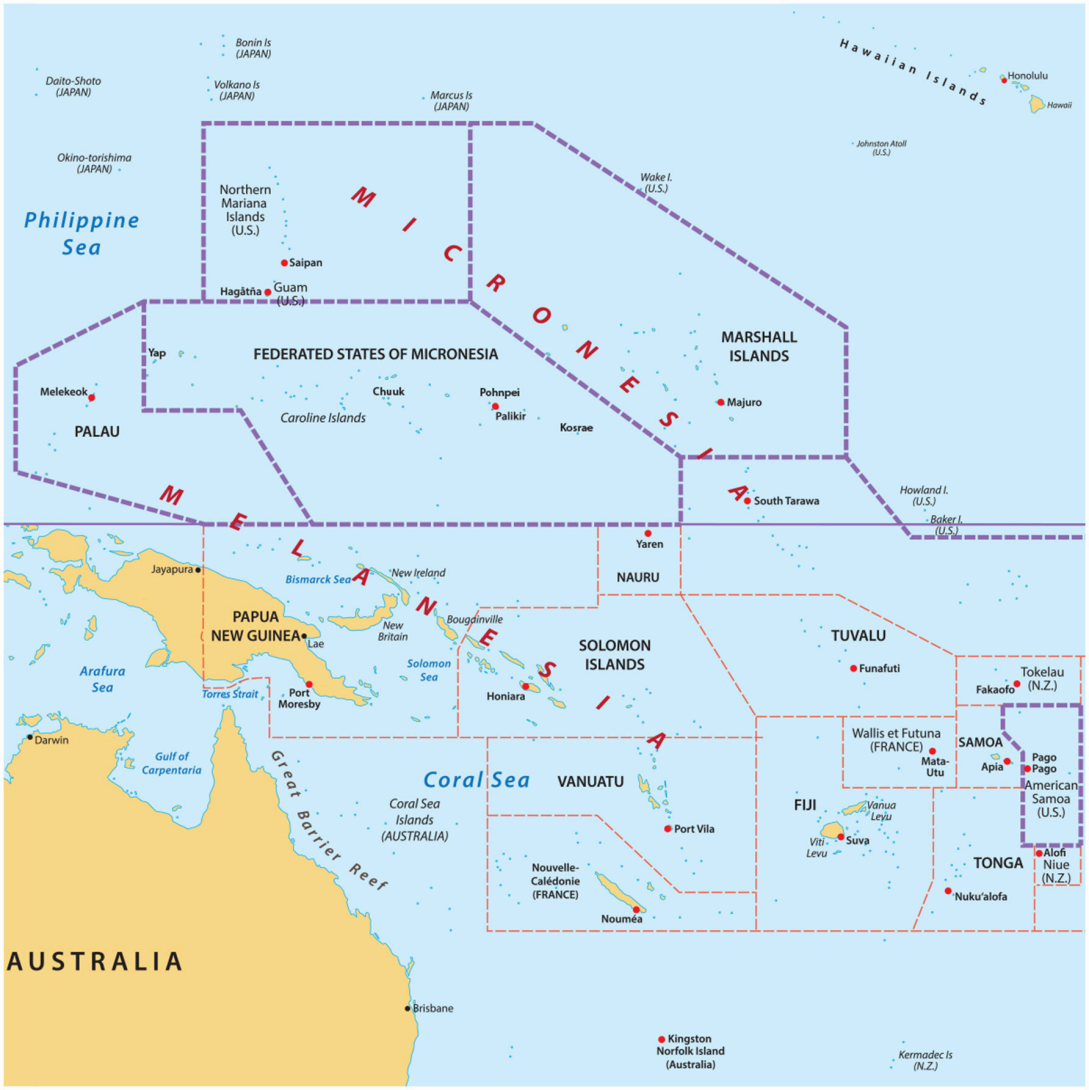
Map of the US Affiliated Pacific Island jurisdictions. https://www.123rf.com/profile_rusak.

**Figure 2:**
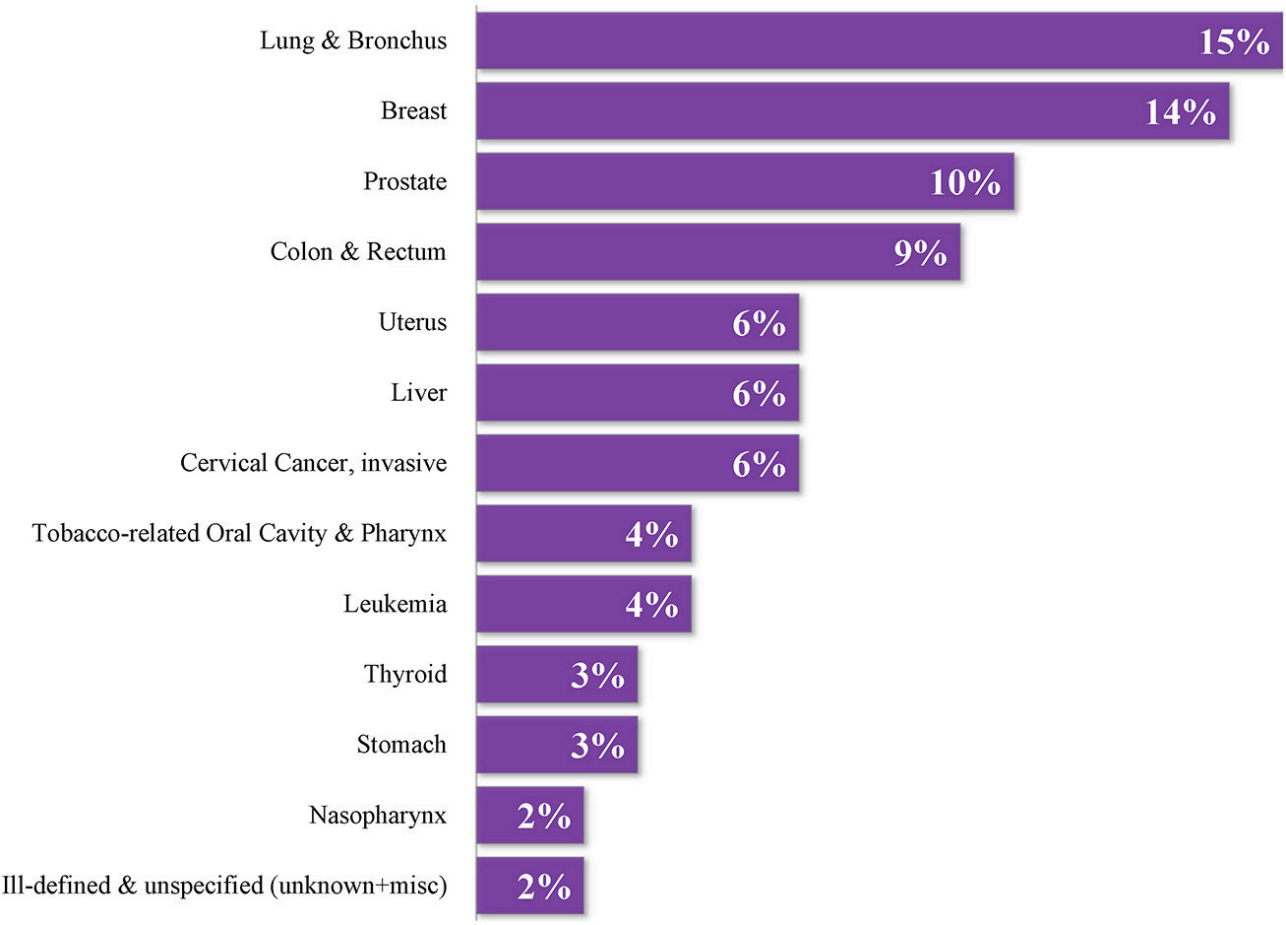
Proportional distribution of adult cancer in the USAPIJ 2007–2015. Courtesy of Pacific Regional Central Cancer Registry, University of Hawaii, 2018.

**Figure 3:**
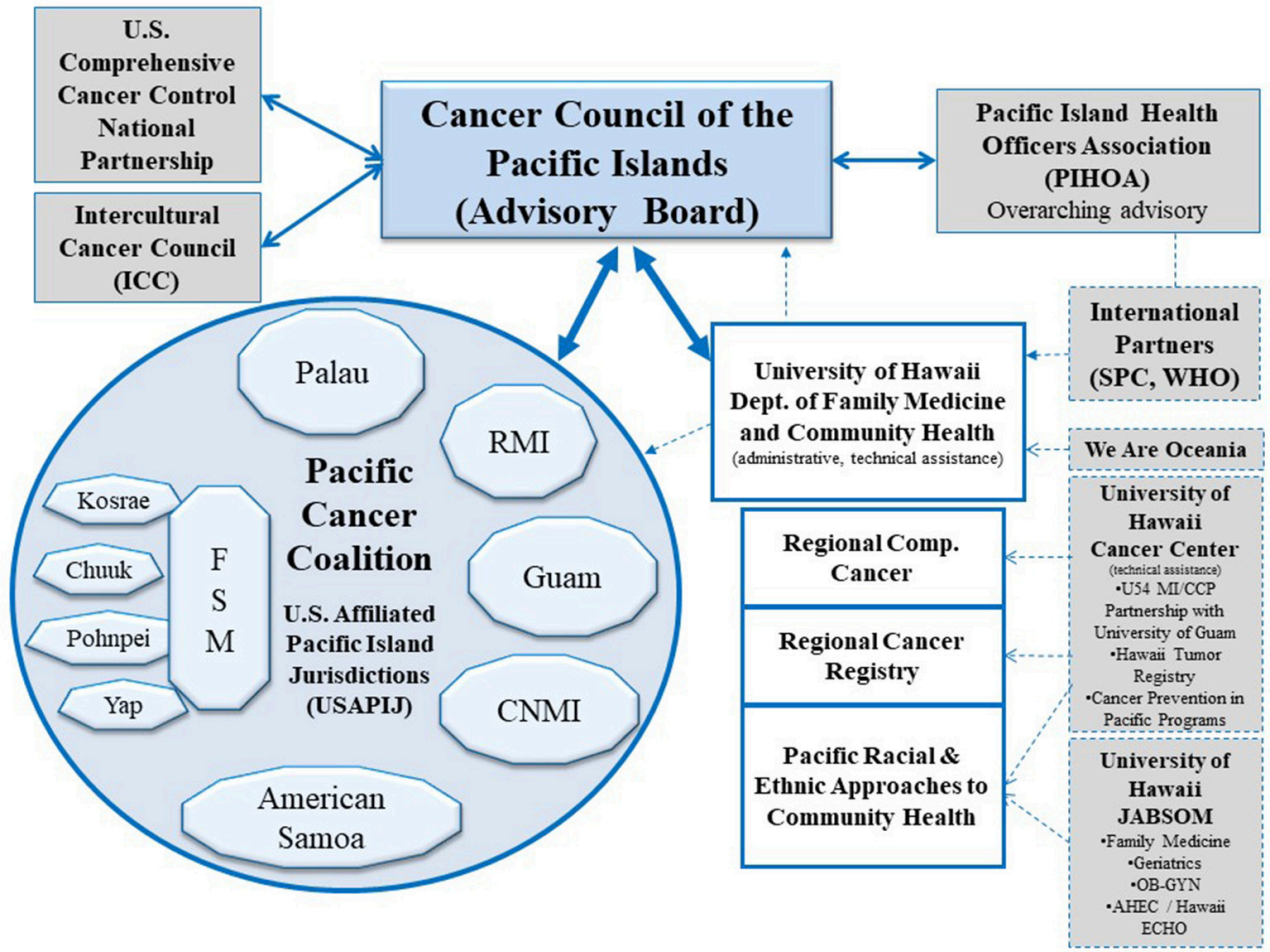
Pacific regional cancer control partners.

The culturally diverse United States Affiliated Pacific Island Jurisdictions (USAPIJ) are politically linked to the United States (US) as US Territories (Guam, American Samoa), a US Commonwealth (Northern Mariana Islands), and through Compacts of Free Association (COFA) (1) [Federated States of Micronesia (FSM), Republic of the Marshall Islands (RMI), Republic of Palau (ROP)]. Figure 1 is a map of the USAPIJ (https://www.123rf.com/profile_rusak). Cervical cancer incidence in the RMI is 7 times the US incidence, liver cancer in Yap FSM is 3 times the US rate (2), mammography services are non-existent in the FSM, and access to cervical cancer screening in many of the COFA nations is limited. The risk factors for many preventable cancers such as obesity (3), tobacco (4), hepatitis B (5), poverty (6), and low education achievement are more prevalent in the USAPIJ compared to the US. Twelve years of nuclear testing in the RMI adds to the cancer burden for the region (7–9). Only Guam has oncology services on the island. Figure 2 describes the burden of cancer throughout the USAPI from 2007–2015. Due to the inadequate screening and on-island diagnostic capability in most jurisdictions, there is underreporting of cancer cases. Nevertheless, high regional prevalence of cervical, oral and liver cancer are noted. To address this, the USAPIJs have organized themselves as individual entities and regionally to manage and address the cancer risks, incidence, and mortality disparities in their respective Pacific environments. This organization is pictorially represented in Figure 3 and is the focus of this community case study.

### USAPI regional cancer health planning question

The USAPIJs are small island nations, geographically dispersed, culturally diverse, have small populations, and many are resource poor. They have significant cancer and other non-communicable disease (NCD) health and health care disparities compared to the US. The USAPIJs were former or are Territories of the US and are currently economically and politically closely linked to the US. How do these island countries address their significant cancer burdens in a relevant and sustainable way in their current socio-economic, cultural, and political environment?

This community case study describes the developmental history, 1997–2017, of the Pacific Regional Cancer Control Partners (PRCP) (Figure 3). The partnership was developed for and by indigenous Pacific peoples of the USAPIJ to manage the cancer risks, morbidity, and mortality in their respective Pacific environments. The study provides an analytical framework to understand the organizational membership and their relationships, how the current partnership unfolded, and how the organization was managed.

### Significance

The socio-ecological framework provides a common language, typology, building blocks, and terms for the PRCP. The institutions, organizations, their linkages, and hierarchy bring form and structure to the PRCP. As the PRCP evolves and adjusts to physical, economic, socio-cultural, and environmental realities in the USAPIJ, a critical analysis of this structure is warranted. Through the PRCP, local cancer coalitions, a regional cancer registry, 12 years of regional cancer control plans, cancer prevention programs and research projects have been implemented. Identifying the elements and a mechanism that leads to productive activities, interactions, and coordination of a multi-coalition, multi-sector organization in this environment is needed. The management body must be able to effectively work within the multi-dimensional partnership, including flexibility to manage rapid changes to the partnership and working relationships in the unique environment of the PRCP. The partnership structure and organization may have important implications for practice in public health and population health. Global cancer prevention and control efforts which are developing or evolving in fragile policy and/or resource-limited environments may benefit from particular management strategies.

### Innovation

There are increasing efforts led by government, healthcare, and academic institution partnerships to address a variety of health concerns, including cancer, in national and international environments. However, there is a dearth of information regarding national and international partnerships for health that are community-centric, multi-level, multi-system, and that are operationalized in a multi-cultural resource-limited context. As the need for better cancer control in frontier resource-limited environments emerges, the characteristics and fundamental building blocks, matrix, and management of existing regional organizations that have demonstrated progress and sustainability (10) would serve to close a significant global information gap. This case study describes developmental factors that led to the PRCP in context of the USAPIJ's physical and socio-cultural environments. This case study of the USAPIJ community is expected to illustrate that a socio-ecologic blueprint for regional coalitions can lead to the formation of a relevant organization and management infrastructure for cancer control.

This case study adds to the knowledge that is already known about processes for effective collaboration (11) for Pacific Island Cancer (12) and Chronic Disease Programs (3) and integrated logic models that describe best practices to ensure program focus on implementation and evaluation. The knowledge gap is about community-centric, multi-national, multi-system organizations to affect cancer prevention and control in resource-limited settings. Is this type of partnership possible and what are the foundational elements?

This topic is important to illustrate how partners and management processes articulate appropriately as cancer risk and prevalence change, or as cancer technology evolves. The operations of the partnership should be sustained in face of changing leadership, personnel, funding, and policy environments.

This case study offers an opportunity to provide a construct that challenges assumptions about the powerlessness of politically isolated communities in low resource settings to manage complex illnesses in complex environments. A new set of recommendations applied to practice in other regions of the globe may be considered.

## Methodology

### PRCP

The community-centered partnership, the PRCP, is the subject of this community case study. The approach is illustrative to establish a common language, terms, typology and building blocks of the PRCP. The analytical framework will describe the relationships between the internal and external partners of PRCP. The analysis framework, the object of the case study, will be the socio-ecological theoretical framework (SEF). The SEF will be mapped to the PRCP in **Figure 5**, to illustrate an organizational structure which is able to systematically address the multiple influences on cancer outcomes in a Pacific Island context.

The PRCP is based and operates in a unique environment. The jurisdictions are all island nations, with limited health resources (13) and have significant challenges across the entire cancer prevention and control continuum.

### History of PRCP and CCPI

The history of the PRCP began in 1993–1997 with USAPIJ clinicians noticing a significant increase in cancer cases presenting to the hospitals and clinics. The apparent rapid increase in cancer cases to health care services was highlighted to policymakers and civil society. Local efforts and associated finances to systematically address the cancer burden were minimal or absent. From 1997, community champions sought partnerships and funding to assess the regional cancer burden and to develop a systematic approach to address the burden. Because of the advocacy, in 2002, the Cancer Council of the Pacific Islands (CCPI) was formed with funding from the US National Cancer Institute health disparities program. The CCPI was charged with defining the USAPIJ's cancer burden and planning a path forward. The CCPI was then composed of a senior public health and a clinical professional representing each USAPIJ and were appointed by the respective Director or Minister of Health (14–16). The secretariat and technical support of the CCPI was provided by the Department of Family Medicine and Community Health from the John A. Burns School of Medicine, University of Hawaii. In 2004, the CCPI with its academic partners were successful in competing for a regional Centers for Disease Control and Prevention (CDC) comprehensive cancer control planning cooperative agreement. By 2007, the CCPI successfully developed community cancer coalitions in each USAPIJ. Each respective coalition successfully competed for jurisdiction-specific CDC comprehensive cancer control implementation cooperative agreements that provided sustainable funding for the CCPI. The CCPI and its partners then successfully competed for a CDC population-based regional cancer registry cooperative agreement and a CDC Center of Excellence in the Elimination of Disparities Racial and Ethnic Approaches to Community Health grant.

The work of the CCPI is to convene the PRCP and develop and operationalize a USAPIJ regional comprehensive plan based on the respective needs of the jurisdiction cancer coalitions. The CCPI provides the administration, leadership, management, and coordination for the PRCP activities. The CCPI activities are funded through a subcontract to the University of Hawaii totaling 7–12% of each respective jurisdictions' CDC-funded comprehensive cancer control cooperative agreement.

### PRCP structure

Figure 3 describes the Pacific Regional Cancer Control Partners (PRCP), the partnership that evolved since 2002 through the work of the CCPI. Collectively, the USAPIJ coalitions comprise the Pacific Cancer Coalition. Figure 3 illustrates the internal structure of the USAPIJ communities where jurisdiction-specific cancer prevention and control occurs through their respective cancer coalitions. The external structure is comprised of the regional, US National and International partners, organizations, and institutions that support the internal Pacific Cancer Coalition. The external structure is composed of relevant policy groups, advisory bodies, funding organizations, and advocacy groups. Together, the collective internal and external structures make up the PRCP. The CCPI is the conduit between the external and internal structure. The CCPI continues to function as the advisory and management organization within the PRCP.

In 2011, a structured self-assessment of the internal and external partners, with respect to cancer control planning objectives and partnership relationships, was conducted. The internal structure was determined to be functioning well and the external structure was functioning satisfactorily (12).

### Socio-ecological framework

The Socio-Ecological Framework (SEF) is a theory-based framework that describes the multi-dimensional and interactive spheres of influence (individual, interpersonal, organizational, community, and policy) that influence health behaviors and outcomes. The spheres of influence often interact in a non-hierarchical and non-linear fashion.

**Figure 4:**
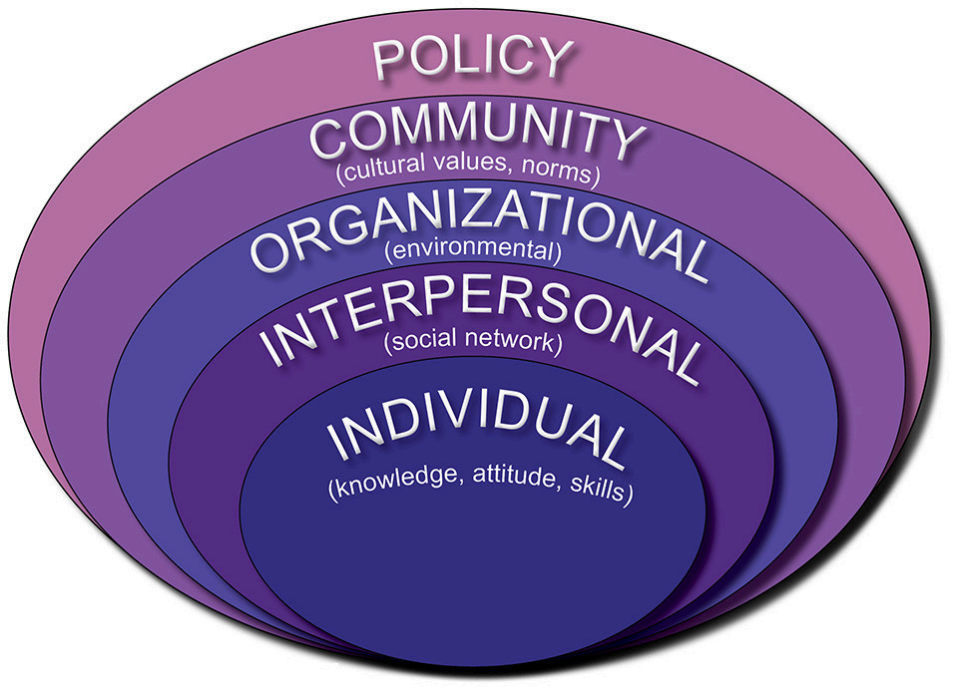
Socio-ecological framework.

The SEF can be used to pinpoint behavioral and organizational leverage points for operationalizing cancer prevention and control plans. Figure 4 is an adaptation of ecological theory as outlined by Grzywacz and Fuqua (17).

In this case study, the SEF was utilized to organize and describe the systems and pathways that interface with cancer prevention and control efforts in the USAPIJ environment. The SEF serves to deconstruct the PRCP to visualize where engagement with the spheres of influence occur. The users and partners of the PRCP could identify their placement and relationship in the PRCP as it relates to the SEF. The SEF provides a method to organize the complex interactions that are associated with cancer etiology, epidemiology, and control.

**Figure 5:**
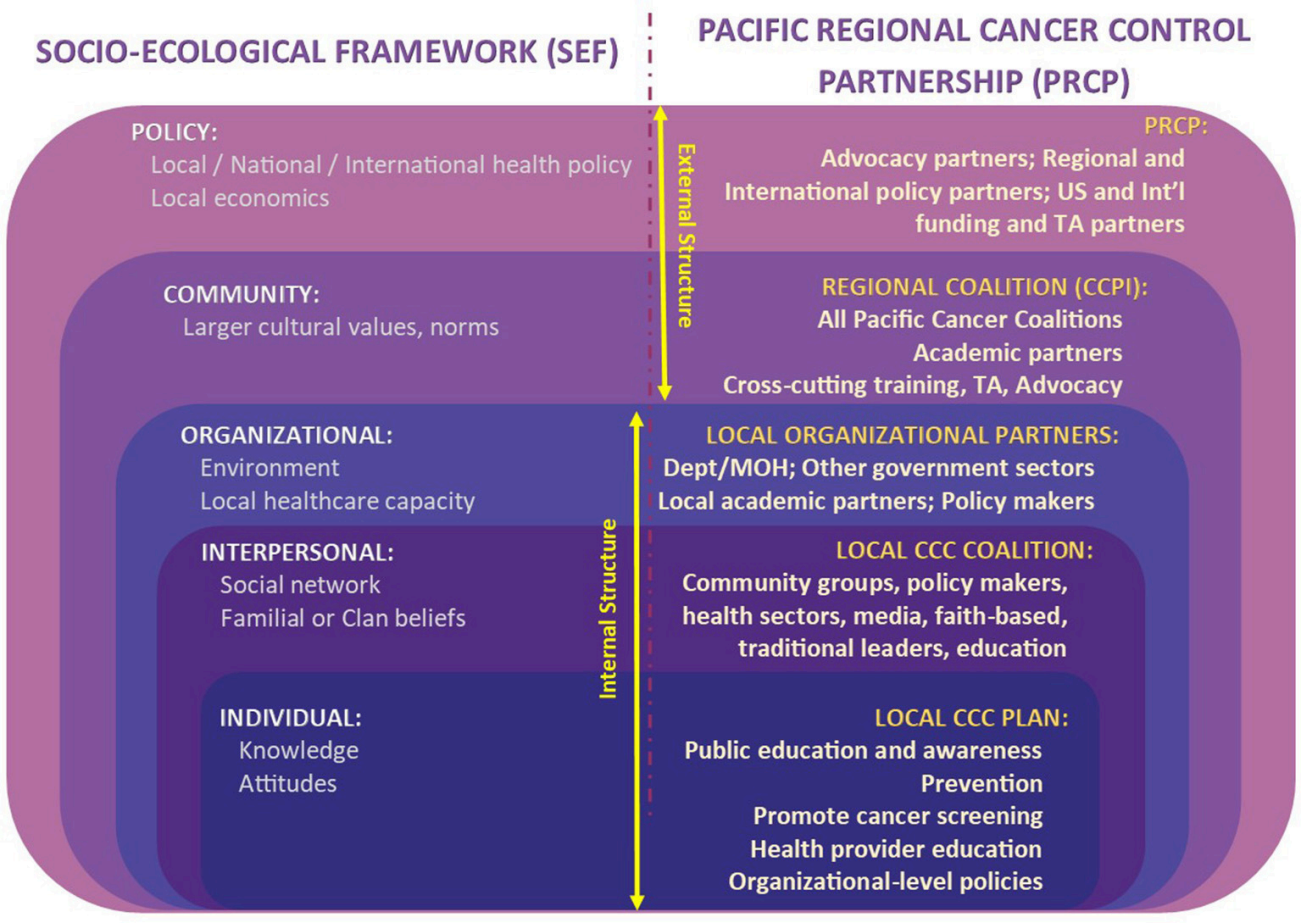
The Pacific regional cancer control partnership within the socio-ecological framework.

The CDC framework for prevention, based on the SEF, notes that a multi-level systems approach with multiple influences is needed to effectively address the continuum of cancer health and health care. An approach to public health prevention and control that uses a combination of interventions at all levels and systems of the model will likely have a higher chance of success (18, 19). The PRCP is an example of a multi-level systems approach to cancer control which has been operationalized in a resource-limited, multi-national setting (Figure 5).

### PRCP through a SEF lens

#### Internal structure (pacific cancer coalition)

There are 10 jurisdiction-specific cancer coalitions within the internal structure. They are Federated States of Micronesia (FSM) National and the four FSM States of Chuuk, Kosrae, Pohnpei, and Yap, the Republic of Palau (ROP), Republic of the Marshall Islands (RMI), Guam, Commonwealth of the Northern Mariana Islands (CNMI), and American Samoa. They form the internal cancer planning structure of the USAPIJ and are noted in Figure 3 as the Pacific Cancer Coalition.

The 10 coalitions have a diverse multi-level, multi-system community memberships including cancer survivors and their family members, national or local government leaders, business leaders, clinicians, public health personnel, educators, faith-based leaders, traditional leaders, and civil society. The work of the country coalition is to develop, operationalize and evaluate their respective jurisdiction comprehensive cancer control plans. Each cancer coalition has a paid coordinator who manages and administers the local coalition. The coordinator and cancer planning expenses, but not the implementation costs, are funded through a CDC cooperative agreement. Community participation through a cancer coalition promotes relevant community engagement and a process driven by community-centered needs.

The coalitions assess their community cancer-related issues, create a cancer plan based on their assessment, prioritize objectives in the plan, and implement the plan using a community-based participatory approach (20). This approach allows individuals and their respective communities to provide input and to become vested in the interventions.

In three jurisdictions (Guam, RMI, and ROP), a non-communicable disease (NCD) coalition is the overarching jurisdiction coalition with a cancer-focused sub-coalition. In all jurisdictions, however, representatives from the CDC-funded NCD programs (i.e., diabetes, tobacco, NCD risk-factor surveillance) participate in the cancer coalition activities. Linking with NCD prevention and control partners and programs leverages limited human and financial resources.

The local community dialogue regarding personal, interpersonal, cultural, organizational (healthcare and education), and policy (government and traditional) influences associated with cancer outcomes, as noted in the SEF, begins with the cancer coalition during their assessment and cancer planning efforts. Through an iterative process, these elements are captured, discussed, and incorporated into the cancer plan by diverse community stakeholders.

Each of the jurisdictions plans are discussed at the regional CCPI meetings to identify cross-cutting themes, to handle unmet needs through economy of scale, and to identify objectives that could be or are influenced at the regional and international levels.

#### External structure

The external structure is where the international, academic, advocacy, and extra-mural funding organizations work to support the planning efforts of the internal structure. The dialogue with the external partners largely occurs at the CCPI, which is composed of USAPI Indigenous representatives. The CCPI develops a USAPI Regional Comprehensive Cancer Control Plan, informed by the jurisdiction cancer plans and their cancer coalitions. The external organizations engaged by the CCPI are those that directly or indirectly influence cancer outcomes in the island jurisdictions. These include organizations that function in the policy, technical assistance, funding, medical, scientific or research domains.

#### Policy organizations

Policy organizations develop, interpret, influence and may determine health relevant policy for the USAPIJ. The PRCP maintains policy engagement at three levels: (1) local governments, (2) national governments, and (3) international governments (US National and global). Local government interactions occur with and within the community coalition, whereas national and international engagement occurs through regional policy organizations. The regional policy organizations are the Pacific Island Health Officers Association (PIHOA), the World Health Organization (WHO), and the Secretariat of the Pacific Community (SPC). PIHOA is a 501(c)(3) regional organization composed of the Ministers and Directors of Health of the USAPIJ. PIHOA provides policy support for all healthcare matters for the USAPIJ region. The WHO and SPC are health policy, developmental, and technical assistance organizations.

#### Technical assistance and funding agencies

Technical assistance and funding agencies include the Centers for Disease Control and Prevention (CDC) and National Cancer Institute (NCI). Through a competitive process, CDC provides cooperative agreements for the Racial and Ethnic Approaches to Community Health (REACH) program, the Pacific Regional Central Cancer Registry, the National Breast and Cervical Cancer Early Detection Program, and the National Comprehensive Cancer Control Program. These cooperative agreements and their associated technical assistance build local capacity for the spectrum of cancer and NCD prevention and control. The NCI provides funding for cancer research and cancer research capacity building through a highly competitive process. International technical assistance and funding is provided through the WHO and SPC, primarily. The FSM, RMI, and ROP nations are also eligible for and leverage resources from foreign Agencies for International Development such as AusAID, NZAID, and several from Asia.

#### Academic partners

The Department of Family Medicine and Community Health (DFMCH) at the John A. Burns School of Medicine, University of Hawaii, as an academic partner, serves as the secretariat for the CCPI. DFMCH provides technical assistance for the CCPI Regional Comprehensive Cancer Control Plan and for 10 Pacific jurisdiction coalition plans. The DFMCH manages the PRCP. The DFMCH leads the CDC's Racial and Ethnic Approaches to Community Health program and the CDC-funded Pacific Regional Central Cancer Registry. These cooperative agreements are leveraged for cancer prevention and control, including cancer risk reduction.

Other academic partners include the University of Hawaii Cancer Center, which develops the cancer research infrastructure in the USAPIJ. The University of Guam partners with specific prevention and research programs.

#### Advocacy partners

There are two US National organizations that are strong advocates for the USAPIJ cancer control. They are: (1) The US Comprehensive Cancer Control National Partnership, a group of 18 national organizations including CDC whose purpose is to support comprehensive cancer control coalitions in the states, tribes, territories, and US Pacific Island Jurisdictions (11) and (2) The Intercultural Cancer Council (ICC), a policy, research, and advocacy organization working on behalf of diverse cancer individuals, their families, and caregivers (21). In 2002, the ICC added the USAPIJ to their mission statement. To date, it continues to promote research, policies, programs, and partnerships to eliminate the unequal burden of cancer among racial and ethnic minorities and the medically underserved in the US, its associated territories, and Tribal Nations.

### PRCP management framework

Appropriate governance and management are important for organizational function. Management is defined as the actions needed to operationalize and carry out the organization's plan. It differs from governance which involves setting strategic goals, defines membership requirements, and sustains financial resources. Governance will not be a topic of this case study.

A management framework may be applied to analysis and typology of the PRCP operations. This case study focuses on the type of management that fits the PRCP operations. Management deals with processes that effectively engage and maintain organizational member interest in shared goals. Management develops methods to monitor progress over time to adjust to change in membership, leadership, or the socio-economic environment.

A subtype of management is adaptive management. Adaptive management is characterized by active consideration of a range of management choices to account for significant uncertainties about the outcome of a single management action (22). There are two general characteristics of adaptive management. Adaptive management requires comparing outcomes of management decisions and the resulting actions. Utilizing data from prior management decisions to develop indicators of progress toward management objectives in a nuanced environment is necessary. Monitoring management results as an iterative process to arrive at better management decisions is the adaptation strategy.

The inclusion of stakeholders affected by management actions in decision-making necessitates a collaborative structure for stakeholder participation and learning. Achieving meaningful stakeholder involvement includes active learning and agreement among participants. Adaptive management includes input from managers, decision-makers, and medical experts and scientists. Equally important, community interest groups and civil society provide crucial and necessary input and guidance throughout the decision-making process. Adaptive management concepts and practices represent innovative, current thinking on resolving conflicting demands and adjusting to changing social, scientific, and political preferences and priorities.

The management function for the overall PRCP resides within the CCPI. An adaptive management strategy is needed and used for decision-making as it applies to the unique circumstances of the internal and external organizations of the PRCP. Each of the Pacific jurisdictions has its own community-based, community-developed comprehensive cancer control plan. The PRCP was formed to serve the needs of the USAPIJ communities. These island countries have limited land mass and the island populations are dispersed over 2 million square miles of ocean with diverse cultural, linguistic, socio-economic, and policy environments.

The overall goal for the USAPIJ is to prevent and control cancer. However, because of the diversity of socio-cultural environments, there are often divergent priorities and strategies around cancer plan objectives. It is unlikely that a single management decision and actions on any given objective would meet the needs of a diverse, dynamic group. The CCPI adapts its management decisions and actions to the respective cultural and policy environment to achieve goals and has learned to adapt quickly to unanticipated responses.

## Discussion

This case study offers an organizational approach to understand, plan, and address complex socio-ecological environments that affect cancer or NCD outcomes. The PRCP model provides a pathway for information input, synthesis, and prioritization based on the SEF spheres of influence at the community level. The community plan is then moved to regional and international partners to help implement those parts of the plan that require external assistance. The external assistance may be policy, funding, research, data, or training support described in regional cancer and control planning.

Success of the PRCP is measured in process outcomes such as active cancer coalitions, comprehensive cancer control plans at the jurisdiction and regional level, acquisition of millions of dollars of funding for planning and implementation efforts, implementation of a regional population-based cancer registry, and sustained efforts of the CCPI and coalitions for 17 years. Developing resource-appropriate cervical cancer screening programs, enhancing nutrition programs, developing exercise programs, and introducing jurisdiction and regional tobacco control policies, and cancer prevention control interventions are all project outcomes. Cancer data is now being collected systematically via a CDC population-based cancer registry to provide baseline cancer metrics. Notably, changes in cancer outcome metrics such as incidence, morbidity, and mortality rates will not be seen for another 15 years due to the chronicity of the illness and ongoing resource-limitations in the various health systems.

### Applications

A PRCP-type organization may be applied in several contexts. The structure provides a format and path for internal community cancer plans to be facilitated by policy, funding, and organizational spheres of influence that are external and relevant to the community. The interface for internal—external discussions is a regional coalition composed of the respective community leaders. Rural, resource-limited, small population communities, environments with diverse cultural groups, and communities with limited human health resource capacity could increase their political visibility, be more competitive for donor or large institutional funding, and work synergistically through economy of scale. The PRCP model could be applied in the multi-national island countries in the South Pacific and Caribbean. The PRCP model could also be utilized in rural communities within a large State or province such as rural communities in the Midwest or southern US, or States within the union coming together to form a US National cancer plan. This model may be also scaled for large countries, whereby a PRCP type model is designed to represent scattered special populations, which identify by age, gender, ethnicity, sexual orientation, or disabilities within a large country; such as cancer in people with disabilities in the US or cancer in the southern villages of Vietnam. Finally, this model could be applied to link large countries with limited resources such as those in Southeast Asia or sub-Saharan Africa, to be competitive in the global environment.

### Limitations and challenges

Whereas this is a community-based, multi-level collaborative, rapid outcomes and quick decision making is not the norm. Acquiring funding and managing funding requirements is a doable but challenging task in a multi-national environment. Maintaining community champions and leaders is difficult in rapidly changing economic and political environments. The planning and work must be supported within the coordinating and advising central regional organization, CCPI, by a dedicated, passionate, and mission-driven secretariat.

Bringing island countries together to form a functioning collaborative took time and effort. Regional collaboration for health or cancer control was not an established method of doing business prior to 2002. Each island jurisdiction historically addressed cancer control in an independent fashion because of unique cultural and sovereign identities. The collaborative began as a concept, driven by an unmet need. There was no funding and no structure from which to work. After 5 years of steady advocacy to funders and US National and international policymakers, the USAPI became more visible. The evidence base about the cancer in the region was sparse and anecdotal. The most supportive organizations were those which understood minority health and health equity, such as the ICC and the NCI cancer health disparities program.

Lack of support and active undermining from funded health programs with content related to cancer control (tobacco, diabetes, nutrition, physical activity) was not unusual. These programs feared that a strong cancer program would be a major competitor for limited funding sources. Multi-sector health coalitions and civil society interests were the force that harmonized many of the NCD health efforts.

Working in a community-centric, community participatory engaged model presents challenges. Health departments, researchers, or funding organizations often desire primary decision-making authority. Regularly scheduled communications to keep health leadership informed and health leader participation in organizational decision making mitigates this issue.

Understanding major factors that enhance or limit the ability to replicate the PRCP organization structure and function is important. Adaptations of parts of the organizational structure and function can be nuanced to a particular context with three essential factors that make the PRCP work.

First, a PRCP type organization operationalizes the SEF theory to benefit the communities in a particular geographic, political, or regional context. The community (internal organization) is the centerpiece and is served by the organization. The product of the organization is delivered in the community. Community engagement occurs with a multi-sector, multi-level, diverse stakeholders collaborative and is evaluated periodically to its form and function. Health care institutions, funding agencies, and government bodies may dominate the conversation and control the organization. The organization works to create a level playing field for all participants and genuinely values the wisdom and work of the community. The organization may consist of a few partners and often begins with only one or two partners. A regional council and a few community coalitions may be the only components of the organization for years.

A second factor is funding. A PRCP like organization and its community partners require an appropriate level of financial support to operate. Most of the work of the organization is in-kind through contribution of time and expertise. Although synergy of work and economy of scale make the work efficient and doable, there must be sustainable operational funds. Essential personnel which should have direct funding support include community coalition leaders and a regional body secretariat. The financial resources may be derived from several sources, including grants (local, national, international), development aid, philanthropic support, project development funding, and government funding/assistance. At the regional level, a dedicated secretariat is needed to assess the regional body's financial needs, identify funding opportunities, write and submit grants, manage organizational funds, and to work intensively with funding agencies.

The third factor is sustainability as it relates to policy and planning. Support from the country level policymakers and health leaders is essential. Work that is undertaken by the local coalitions and regional body should involve policymakers and health leaders. Output from the local coalitions should directly benefit their respective communities, augment the local health plan, and fill an unmet local need. Sustainability strategies should be included in the comprehensive cancer planning prevention and control process. As with any effort that galvanizes institutions and communities, a long view toward success and sustainability must be part of the plan.

## Conclusion

The PRCP, a multi-level, multi-system, multi-cultural partnership to address the cancer burden in the USAPIJ evolved over 20 years (1997–2017). A SEF has been used to identify and categorize the systems and organizations that interface with cancer prevention and control in the USAPIJ. The PRCP has an adaptive management strategy which best fits community-centric coalitions requiring participatory engagement for decision making. The PRCP form fits its function to address the heavy burden of cancer in the USAPIJs. This case study addresses a knowledge gap through an example of a partnership, informed by the SEF, that has been able to sustain and operationalize comprehensive cancer control plans in a highly nuanced, resource-limited environment. The SEF provides a blueprint for the PRCP organizational structure and a mechanism for its function. The PRCP concept, a community-centric model for cancer control in multi-national resource-limited environments, may be scaled to other global environments.

### Recommendations for organizational development and sustainability

1. Developmental partners should understand the SEF and how the levels of the SEF influence cancer outcomes
2. Community well-being should be at the center of all work
3. Maintaining functioning multi-sector cancer community coalitions to develop local cancer prevention and control plans is essential
4. Regional advisory/coordinating organization should have equal representation from informed leaders from the collaborating communities who are appointed by their respective Health Ministers or Directors
5. The Regional advisory organization develops a regional cancer control plan which is informed by the local community plans
6. Developing leadership capacity and promoting health equity must be a core value to promote sustainability
7. Periodic evaluation of the functioning of the internal and external organization is essential
8. Periodic evaluation of the community and regional cancer planning process and outcomes is essential

## Author contributions

NP and LB-L coordinated the writing of the manuscript. NP, LT, SR, LB-L, MR, and JT contributed to the design. JT, HG, MR, LT, NP, and LB-L contributed to the concept. NP wrote the first draft of the manuscript and is the corresponding author. MN and LB-L wrote sections of the manuscripts. All authors contributed to manuscript revisions, read, and approved the submitted version.

### Conflict of interest statement

The following authors' (NP, MR, JT, MN, HG, SR, LT, LB-L) institutions received CDC cooperative agreements for their work on the comprehensive cancer control, the REACH program, or cancer registry programs. The NCI CRCHD U54 mechanism supported cancer research initiatives.
